# Early toxicity and treatment outcomes of extended field-intensity modulated radiotherapy for cervical cancer patients with para-aortic nodal metastasis

**DOI:** 10.3332/ecancer.2019.957

**Published:** 2019-08-06

**Authors:** Meetakshi Gupta, Supriya Chopra, Shreya Kunder, A Dheera, Devaraju Sampathirao, Reena Engineer, Jaya Ghosh, Lavanya Gurram, Umesh Mahantshetty, Sudeep Gupta, Shyam Shrivastava

**Affiliations:** 1 Department of Radiation Oncology, Tata Memorial Centre, Homi Bhabha National Institute, Parel, Mumbai, India; 2 Department of Radiation Oncology, Advanced Centre for Treatment Research and Education in Cancer, Tata Memorial Centre, Homi Bhabha National Institute, Kharghar, Navi Mumbai, India; 3 Department of Medical Physics, Tata Memorial Hospital, Tata Memorial Centre, Homi Bhabha National Institute, Parel, Mumbai, India; 4 Department of Medical Oncology, Advanced Centre for Treatment Research and Education in Cancer, Tata Memorial Centre, Homi Bhabha National Institute, Kharghar, Navi Mumbai, India

**Keywords:** cervical cancer, PALN, EFRT, IMRT, toxicity

## Abstract

**Objective:**

Extended-field radiotherapy (EFRT) with concurrent chemotherapy represents standard treatment in cervical cancer patients with para-aortic lymph nodal (PALN) metastasis. While EFRT with Intensity Modulated RT (IMRT) has been demonstrated to reduce toxicities, the dose thresholds for minimizing acute toxicity is not clear. The present study was undertaken to report the early toxicity with extended-field intensity-modulated radiotherapy (EF-IMRT) for carcinoma of the cervix in our cohort of patients and determine dose-volume parameters that predict ≥grade II haematological toxicity and diarrhoea.

**Methodology:**

This was a retrospective study of consecutive cervical cancer patients with PALN metastasis treated with EF-IMRT. Patients received rotational IMRT +/- neoadjuvant chemotherapy (NACT) and/or concurrent chemotherapy (45–50 Gy/25#/5 weeks) followed by high-dose rate brachytherapy. Acute haematological and gastrointestinal toxicity (diarrhoea and vomiting) was correlated with doses received by bowel and marrow. Receiver operator characteristics curves were used for deriving thresholds that predict for increased toxicity and tested on univariate and multivariate analysis. Finally, disease free and overall survival (DFS and OS) was calculated.

**Results:**

A total of 43 patients were included. One-fourth of the patients (11/43) received NACT and 88% received concurrent chemotherapy. Within the upfront EF-IMRT cohort, 22.6% and 9.7% patients developed grade ≥III haematological (HT) and gastrointestinal (GI) toxicity respectively, with an increase in HT (≥ grade III HT =67%) in patients receiving NACT (p = 0.007). In the entire cohort bone marrow Volume receiving 10 Gy (V10>) 90% correlated with an increase in ≥ grade III HT (p = 0.05). No dose volume thresholds could be validated for GI toxicity. The OS and DFS at 2 years was 56% and 54%, respectively.

**Conclusion:**

EF-IMRT is a feasible option for cervical cancer patients with PALN involvement and is associated with acceptable grade III toxicity. Future studies need to focus on minimizing HT toxicity.

## Background

Cervical cancer is the second most common cancer in women in the developing world. Standard of care includes pelvic radiotherapy with concurrent cisplatin. The pattern of spread in cervical cancer is orderly, initially involving the lower pelvic lymph nodes and then progressing to higher pelvic lymph nodes and para-aortic lymph nodes (PALN) [1] The Gynecology Oncology Group reported PALN disease incidence to be 5% of stage I, 16% stage II and 25% stage III patients [1]. F-18-fluoro-2-deoxy-D-glucose (FDG)-positron emission tomography/computed tomography (PET/CT) has been shown to be a more sensitive method than CT for detection of PALN in patients with cervical carcinoma and abnormal FDG uptake has been seen in around 21% patients through all stages [2]. Although PALN involvement is a poor prognostic factor and reduces the long-term survival, select patients can potentially become long-term survivors following locoregional radiotherapy. Extended-field radiotherapy (EFRT) with concurrent chemotherapy, therefore, currently represents standard recommendation in patients presenting with cervical cancer and with PALN metastasis [3]. Traditionally, EFRT has been delivered with parallel opposed or conformal fields. Conventional radiotherapy technique for EFRT includes irradiation of large volumes of small bowel and bone marrow, leading to significantly increased gastrointestinal and haematological toxicities. Addition of concurrent chemotherapy further exacerbates this toxicity. Use of radiotherapy (hyper fractionated or standard) and concurrent chemotherapy was associated with 83% and 41% incidence of acute and late grade II or higher toxicity. The Radiation Therapy Oncology Group (RTOG) 9201 study also reported a very high incidence of acute and late toxicities with the use of hyperfractionation while using conventional radiation portals [4, 5].

Intensity Modulated Radiotherapy (IMRT) provides higher conformity of the high dose volume for the extended targets and reduces the dose to organs at risk (OAR), thus leading to reduced acute and late toxicity. While EFRT with IMRT is demonstrated to be associated with reduced acute and late gastrointestinal and marrow toxicities [3–5], the dose thresholds for reducing gastrointestinal and marrow toxicities are not very clear. Data from pelvic radiation demonstrates that both high and low doses received by bowel and marrow may be important in reducing haematological and bowel toxicities [6].

In our institution Image-guided, rotational intensity modulated radiotherapy has been increasingly utilised for extended-field intensity-modulated radiotherapy (EF-IMRT) for optimizing therapeutic ratio through optimal coverage of target, nodal dose escalation and to minimize dose to adjacent organs at risk. The present study was undertaken to report the early toxicity with EF-IMRT for carcinoma of the cervix in our cohort of patients and determine dose-volume parameters that predict ≥grade 2 haematological and diarrhoea.

## Methodology

After institutional review board approval, all consecutive cervical cancer patients treated at Tata Memorial Centre, Mumbai, India, receiving EF-IMRT from between 1^st^ January 2013 to 31^st^ December 2017, with or without neoadjuvant and/or concurrent chemotherapy, were included. Case records of all patients were examined and information regarding patients’ performance status, local stage, histology, the extent of nodal involvement and treatment were recorded.

### Treatment policy

For administering EFRT, our institutional policy mandated verifying the involvement of PALN through histological or cytological diagnosis. In patients wherein a tissue diagnosis was not feasible a combination of clinical radiological findings (like PALN >10 mm in short axis or uptake on positron emission tomography in presence of pelvic lymph nodes) was considered as indicative of PALN involvement. Therapeutic management of these patients was decided in a multidisciplinary joint clinic. Patients with small nodes < 3 cm in size below the renal hilum were considered for upfront EFRT with concurrent chemotherapy (cisplatin 40 mg/m^2^) and brachytherapy. However, those with large> 3 cm nodes or nodal conglomerate or nodes above the renal hilum were considered for 2–4 cycles of neoadjuvant chemotherapy (paclitaxel 175 mg/m^2^ and carboplatin AUC 6) repeated every three weekly followed by imaging for response assessment. This was followed by EFRT with concurrent chemotherapy and brachytherapy.

### Radiotherapy details

The EF-IMRT planning incorporated CT-based simulation in supine position with knee rest. Scans were obtained after intravenous contrast from tracheal bifurcation until mid-thigh with interslice distance of 5 mm. The primary clinical target volume (CTV) included the entire gross tumour plus the cervix, uterus, parametria and proximal half of the vagina (unless in cases with vaginal involvement, in which the entire vagina was included). In postoperative cases, upper one-third of the vagina and bilateral parametrium was included. This was expanded by 10 mm in the superoinferior and anteroposterior direction and 5 mm in mediolateral direction to generate planning target volume (PTV) for the primary. The lymph node CTV included the entire para-aortic nodal region extending superiorly from D12-L1 vertebral junction to the bilateral common iliac, external/internal iliac, upper presacral (from S1 to S2), and obturator regions. In addition, grossly enlarged nodes >2 cm were delineated separately for nodal simultaneous integrated or sequential boost. A separate margin of 5 mm was generated to generate CTV for a nodal boost. Additionally, a 5-mm margin was given to generate nodal PTV. Rotational IMRT was delivered either with Tomotherapy or Volumetric Modulated Arc Therapy. While the PALN PTV received 45 Gy/25#, the pelvic PTV received 50 Gy/25#/5 weeks. Patients undergoing nodal simultaneous integrated boost received 52.5–55 Gy/25# to gross nodes while those undergoing sequential boost received an additional 6–8 Gy/3–4 fractions. The decision to implement a concurrent or sequential boost was physician dependent. Organs at risk delineation included rectum, bladder, femoral heads, bowel bag, duodenum, kidneys and spinal cord. Bone marrow was delineated retrospectively and included entire vertebral bodies from D10-L5, sacrum, coccyx, ilium, ischium, pubis, femoral heads and upper third of the femur. While ensuring that 95% PTV was covered by 95% of the prescription dose, all care was taken to restrict mean dose to kidneys <12 Gy, Spinal cord maximum dose <45 Gy, Bladder V40 < 75% and Rectum V40 < 85%. As there were no verified dose volume constraints for bowel and bone marrow for EF-IMRT all efforts were made to minimize low dose spillage (V15 Gy for bowel bag) in the abdominal cavity. The duodenal dose was carefully evaluated especially in case of overlap of nodal SIB. In all cases, care was taken that V55 Gy of the duodenum was <15 cc. No specific dose constraints were used for bone marrow. Image guidance was used for all patients using cone beam CT.

After completing EF-IMRT, patients received HDR brachytherapy to a dose of 7Gy × 3 fractions prescribed to point A. Select patients with disease extending beyond point A received combined intracavitary and interstitial radiation. The planning aim was to deliver 78-84 Gy to point A.

All patients were reviewed weekly during treatment to assess acute toxicity. Toxicity was graded using RTOG scale [7] for gastrointestinal (GI) toxicity and CTCAE version 4.0 for haematological toxicity [8]. Weekly blood investigations were done for patients receiving concurrent chemotherapy. After treatment conclusion, the first follow up was done at 6 weeks to assess acute toxicity and disease response. Regular follow up was done, thereafter 3 monthly for the first 2 years and 6 monthly till the next 5 years and annually thereafter. A response CT imaging was done to assess nodal response at first follow up. Subsequent follow up included clinical evaluation and follow up imaging at the discretion of treating physician.

## Statistics

The primary objective of this study was to evaluate acute toxicity (documented up to 90 days from treatment completion) and it’s correlation with dose-volume metrics. Receiver operator characteristics (ROC) curve analysis was performed to evaluate the area under the curve between dose received by bowel bag and marrow and acute haematological and gastrointestinal toxicity (diarrhoea), respectively. The cut-off thresholds for predicting toxicity were identified as the best fit between high specificity and moderate to high sensitivity for prediction of toxicity and used for univariate analysis. Impact of patient and treatment-related factors was determined on acute gastrointestinal and haematological toxicity.

Disease free survival (DFS) and overall survival (OS) were reported from the date of registration of the patient at the institute and impact of various parameters on OS (such as age, stage, PA nodal disease bulk, location of PA nodes, use of NACT and dose to point A) was analysed. Kaplan–Meir method was used for analysing DFS and OS using IBM SPSS Statistics for Windows (Version 21.0. Armonk, NY: IBM Corp.). The log-rank test was used to evaluate the impact of prognostic factors on outcomes. Multivariate analysis was done using Cox proportional Hazards. *p* value < 0.05 was considered as statistically significant.

## Results

### Patient characteristics

A total of 43 patients were eligible for the study. All were treated with EF-IMRT. All patients in this cohort had pelvic and PALN metastasis. The mean size of the PALN was 1.5 cm and most patients had multiple PALN. Most of the nodes were situated below the renal hilum (90%). None of the patients had non-regional lymph node involvement or distant metastasis at presentation.

Overall, 11/43 patients received NACT followed by EFRT+/- concurrent chemotherapy and 38/43 patients received EF-IMRT and concurrent chemotherapy. Five patients received EF-IMRT alone either due to toxicity after NACT (*n* = 1) or due to contraindications for concurrent chemotherapy (*n* = 4). The baseline characteristics of all the patients, lymph nodal characteristics and treatment details are summarised in Table 1. The dose received by target and organs at risk during IMRT is summarised in Table 2.

### Compliance and toxicity

A total of 38/43 patients received EF-IMRT with concurrent chemotherapy. Of these 43 patients, 32 received upfront EF-IMRT and 11 received after NACT.

### Upfront EFRT cohort

Of the 32 patients receiving upfront EF-IMRT, 28 received concurrent chemotherapy. Of these, 89% (*n* = 25) patients received at least four cycles of concurrent chemotherapy. The median OTT was 8 weeks (7–11 weeks). Three patients had OTT >10 weeks due to treatment interruption owing to varicella infection, thrombotic episode and infective diarrhoea, respectively. All patients completed the planned EF-IMRT. Within the upfront EF-IMRT cohort, 22.6% and 9.7% of patients developed grade III or higher haematological or gastrointestinal toxicity (vomiting or diarrhoea). All except for three patients proceeded to receive brachytherapy. One patient developed distant metastasis before brachytherapy and another two had infective complications and could not get fitness for brachytherapy.

### NACT and EFRT cohort

Eleven patients received NACT followed by EF-IMRT and concurrent chemotherapy. Median PALN size in this subgroup was 2 cm (±0.78). Most patients receiving NACT had multiple PALN (67%). The decision was based on physician discretion as there was no correlation between the size of the PALN and probability of receiving NACT (*p* = 0.157). The most common regimen used was 3 weekly paclitaxel (175 mg/m^2^) and carboplatin (AUC6), with a median of three cycles (range: 1–3). All patients showed at least a partial response to NACT in PALN. More than half of the patients developed ≥ grade II neutropenia on NACT and 1 patient had grade 4 neutropenia and could not be given any further chemotherapy. Therefore, within the NACT cohort, at least half of the patient population had pre-existing grade II haematological toxicity before initiating EFRT + concurrent chemotherapy. Of the entire cohort, 10/11 patients proceeded to receive concurrent chemotherapy. During radiotherapy 5/11 patients required admission and treatment break due to grade 3 diarrhoea. The mean treatment break was for 6 days (range: 4–11). Nine patients required G-CSF support and eight required blood transfusion during RT. Initiation of brachytherapy was not delayed in any of the patients after EFRT completion and most of the break was encountered during external radiation.

The comparative toxicity between NACT and upfront IMRT cohort is depicted in Table 3. The comparative analysis demonstrates a statistically significant increase in grade III leucopenia, neutropenia and any grade III haematological toxicity in patients undergoing NACT before EF-IMRT and concurrent chemotherapy.

### Dose-volume parameters

Details of Bone marrow doses received in the study cohort are summarised in Table 4. A cut-off of 75% and 90% for the volume of marrow receiving 10 Gy (V10) and 65% and 75% for the volume of marrow receiving 20 Gy (V20) was used for analysis as per ROC analysis and data available for pelvic radiation from INTERTECC protocol [9]. V10 > 90% was associated with higher ≥ grade 2 HT (85% versus 35%; *p* = 0.05) and V10 > 75% was associated with a trend towards higher anaemia (73% versus 44%; *p* = 0.09). After excluding the patients receiving NACT, V10 > 75% was associated with significantly higher ≥ grade 2 anaemia (72% versus 16%; *p* = 0.01).

For bowel toxicity, ROC cut-off of V45 bowel bag of 300 cubic centimetres (cc) and V40 bowel bag of 500cc was chosen. This cut-off did not influence ≥ grade 2 GI toxicity. V45 of 200 ccs, V40 of 250 ccs and V30 of 500 ccs also failed to predict increase acute GI toxicity. Hence, no recommendations for dose constraints for restricting acute gastrointestinal toxicity could be reached for bowel doses. Table 4 shows the number of patients exceeding doses as per the ROC cut-offs used. However, it must be noted that none of the bone marrow constraints were prospectively applied but have been achieved during routine clinical planning of EF-IMRT.

### Response to chemo-radiation

Thirty-seven patients achieved complete response at the site of primary on first follow up post radiotherapy. This included patients who received adjuvant postoperative RT. Around 50% of patients achieved a complete response in the PALN and 90% achieved at least a partial response at PALN with (chemo)-radiotherapy. The median follow of the group was 12 months (range: 3–32). At last follow up, pelvic control rate was 93% and PALN control rate was 93%.

### Sites of recurrence

**Figure 1.**
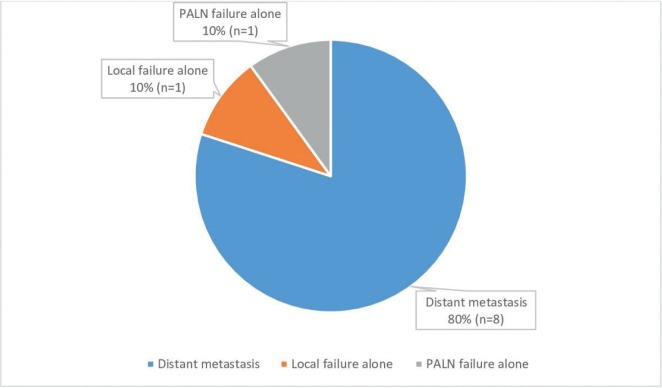
Patterns of failure.

Majority of the patients failed distally (*n* = 8). Most patients failed at multiple sites distally, including lungs (*n* = 3), mediastinum (*n* = 2), para-aortic region (*n* = 2), supraclavicular fossa (*n* = 2), peritoneum (*n* = 1) and bones (*n* = 1). Only one patient failed locally without DM and one patient failed in the treated PA nodal field without DM. Most patients with PALN failure also had multiple other sites of distant failure. Figure 1 shows patterns of failure of the whole cohort. The median disease free survival was not reached for this group.

### Survival analysis

**Figure 2.**
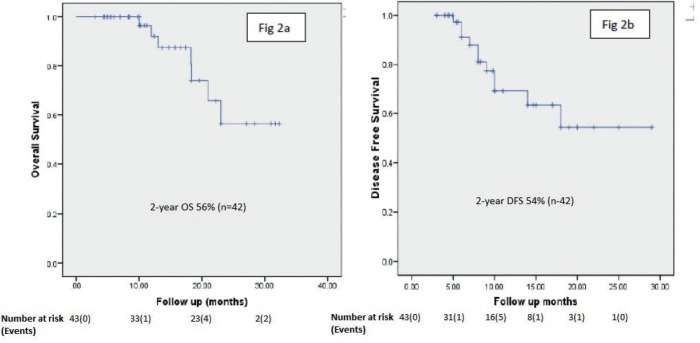
(a) and (b) demonstrate overall survival and disease-free survival of the entire cohort.

The OS and DFS at 2 years was 56% and 54%. respectively. Figure 2a and b show the Kaplan–Meier curves for OS and DFS, respectively.

**Figure 3.**
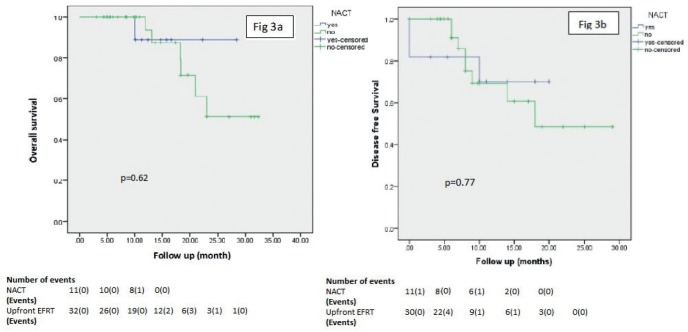
(a) and (b) demonstrate a comparison between overall survival and disease-free survival in patients receiving NACT followed by EFRT± chemotherapy vs upfront EFRT± chemotherapy.

The OS and DFS at 2 years for patients receiving NACT and EFRT was 89% and 67%, respectively and that for upfront EF IMRT was 51% and 51%, respectively (Figure 3a and b). Figure 3 shows a comparison between OS and DFS in NACT and no NACT group (*p* = 0.62 and *p* = 0.77, respectively).

### Prognostic factors

On univariate analysis, FIGO (International Federation of Gynecology and Obstetrics) staging, histology, laterality of the PALN, number of PALN, largest PALN size > 1.5 cm, NACT, and concurrent chemotherapy were not significant predictors of OS. Patients with PALN above the renal hilum had a significantly poorer OS (*p* < 0.001). Multivariate analysis was hence not performed.

## Discussion

EFRT with concurrent chemotherapy remains the standard of care for cervical cancer in patients presenting with PALN involvement and is associated with a 2-year survival of 46-60% [10]. Due to high rates of distant metastasis in this cohort intensification of systemic chemotherapy needs evaluation. However, the high rates of > grade III haematological and gastrointestinal toxicity (up to 80%) jeopardizes patients tolerance to this intensive treatment and may result in prolonged treatment time and high rate of late toxicity and leading to inferior clinical outcome [10]. Strategies for reducing acute toxicity like hyperfractionated RT [11] or the addition of Amifostine to EFRT [10] have led to either exaggerated or similar toxicity without improvement in outcomes.

In recent years IMRT has been evaluated for reduction of acute toxicity during EFRT by potentially reducing the doses delivered to the bowel and bone marrow. A combination of external beam radiotherapy and brachytherapy with concurrent cisplatin has shown to have a 3-year OS of 36-65% (Table 5) with grade III/IV toxicity of 10%–50% with higher survival in series using postoperative extended field radiation or those using para-aortic radiation for positive common iliac nodes. Although IMRT has been used since last decade for improving the tolerability of EF-IMRT, the diverse rates of observed toxicity amongst different series may be indicative of lack of robust guidelines for bone marrow and GI sparing while employing extended fields. While research has been directed to determine the dose-volume constraints for these organs in pelvic radiotherapy delivered with IMRT, little has been published about the appropriate constraints for delivering EF-IMRT in cervical cancer patients. Considering the fact that these patients have a significantly larger volume of bowel and bone marrow irradiated within the RT fields, dose constraints tailored for this subgroup need to be determined.

The present study was therefore undertaken to determine relationship if any with volumes of marrow and bowel irradiated in patients undergoing EF-IMRT. The rate of grade ≥III acute haematological toxicity in our series was 22.6% and 9.7% in those receiving upfront EF-IMRT and concurrent chemotherapy. These are similar to the recently published series with IMRT (Table 5).

We observed that Bone Marrow V10 > 90% was significantly associated with higher ≥ grade 2 HT (*p* =0.04). After excluding the patients receiving NACT, V10> 75% was still associated with significantly higher ≥ grade 2 anaemia suggesting that prospectively applying constraints to bone marrow may help minimize haematological toxicity. Further research is being focussed to answer whether reducing the pelvic bone marrow radiation dose can reduce hematologic toxicity and permit better chemotherapy delivery in patients undergoing CRT. INTERTECC-2 is the first prospective controlled study to test the hypothesis and found that, compared with CT-based bone marrow-sparing IMRT, PET IG-IMRT is more effective in reducing HT rates, which is consistent with previous modelling studies [6]. (F-18) FLT PET is a promising imaging modality that has been used to characterize bone marrow activity and may help in overcoming the limitation of identifying and locating functional bone marrow.

Unlike for pelvic RT wherein there is a correlation of volume of bowel irradiated and acute GI toxicity [12], in the present study we could not validate any of the bowel thresholds, i.e., volume irradiated by 15–40 Gy. While we also tested dose constraints proposed in EMBRACE II study protocol [13], they could not be validated. This could be attributed to the difference in the upper extent of irradiated volume (up to renal vein vs. D12/L1 junction) and the methodology of delineating bowel bag. Determining bowel dose thresholds, therefore, would represent one of the future research for this cohort.

Though not statistically significant patients in NACT cohort had a trend towards better OS and DFS in our cohort and integration of systemic chemotherapy requires further evaluation.

Also as in our series and other published series haematological toxicity outweighs GI toxicity focusing on prospectively studying the dose-volume relationship of marrow irradiation and acute toxicity is desirable. Also, more focussed marrow delineation techniques using FLT-PET may be desirable. While the strength of our study is an attempt to find a dose volume correlation for acute haematological toxicity, we do recognise this as a retrospective analysis consisting of a heterogeneous patient population. A prospective study with a larger number of patients is needed to improve understanding of the dose-volume parameters and the role of NACT in this cohort.

## Conclusion

EF-IMRT is a feasible option for cervical cancer patients with involvement of PALN at presentation and is associated with acceptable haematological and GI grade III toxicity. Further reduction in haematological toxicity may be feasible by restricting the volume of bone marrow receiving 10 Gy between 75%–90%; however, the dose-response relationship for gastrointestinal toxicity including diarrhoea could not be demonstrated. Future studies need to focus on strategies to minimize toxicity to facilitate the integration of systemic chemotherapy for high risk populations.

## Conflicts of interest

None of the authors have any conflicts of interest to disclose.

## Funding

The authors did not receive any funding for the project.

## Institutional review

Institutional Ethics Committee approval was obtained for the study.

## Figures and Tables

**Table 1.** Baseline patient characteristics, lymph nodal characteristics and treatment details.

Median age	55 (±8) years
Previous hysterectomy	4 (8%)
Stage at presentation: FIGO	
Ib1	2 (5%)
Ib2	2 (5%)
IIb	18 (42%)
IIIb	21 (48%)
Histology	
Squamous	32 (75%)
Adenocarcinoma	10 (23%)
Others	1 (2%)
Lymph nodal characteristics	
Pelvic nodal status	
Present	43 (100%)
Mean size of pelvic node/s: (cm)	2.2 (±1.2)
PALN diagnosed on	
FNA and radiology	10 (23%)
Radiology alone	31 (72%)
PET/CT scan	2 (5%)
No. of PALN	
Single	7 (16%)
Multiple	36 (84%)
Mean size of PALN: (cm)	1.5 (±0.6)
Laterality of PALN	
Left	19 (45%)
Right	5 (10%)
Bilateral	19 (45%)
Position of PALN	
Below renal hilum	39 (90%)
Above renal hilum	4 (10%)
Type of treatment received	
NACT+ EFRT± conc. CT	11 (25%)
EFRT± conc. CT	32 (75%)
Brachytherapy	29/32 (Upfront EFRT group) 12/12 (NACT group)
NACT drugs	
P+C	11
Others	1
Median no. of NACT cycles	3 (range: 1–5)
Concurrent chemotherapy	
CDDP	37 (97%)
Others	1 (3%)
Median no. of concurrent CT cycles	4 (range: 2–6)
EFRT doses	
45Gy	25 (58%)
45Gy+ sequential boost (5.4Gy)	10 (23%)
SIB (52.5-55Gy)	8 (19%)
Radiotherapy technique	
Static field IMRT	5 (12%)
Rotational IMRT	38 (88%)
SIB	7 (16%)
Sequential boost	36 (84%)
Mean dose to point A:	75Gy (±8Gy)

PALN, paraaortic lymph nodes; PET/CT, positron emission tomography/ computed tomography; FNA, fine needle aspiration; NACT, neoadjuvant chemotherapy; conc. CT, concurrent chemotherapy; EFRT, extended field radiotherapy; P+C, Paclitaxel+carboplatin; CDDP, cis-diamminedichloridoplatinum; IMRT, Intensity modulated radiotherapy; SIB, Simultaneous integrated boost

**Table 2.** Doses to organs at risk (OAR).

Mean rectum dose (Dmax) (EBRT+BT)	70Gy (±5.4)
Mean bladder dose (Dmax) (EBRT+BT)	80Gy (±8.9)
Mean sigmoid dose (Dmax) (EBRT+BT)	70Gy (±6.3)
Median V45 bowel (cc)	256 (±166)
Median V45 bowel (%)	8.8 (±4.8)
Median V40 bowel (cc)	457 (±184)
Median V40 bowel (%)	14.4 (±6.3)
Median V30 bowel (cc)	850 (±282)
Median V30 bowel (%)	28 (±10.2)
Median V15 bowel (cc)	2205 (±699)
Median V15 bowel (%)	80 (±15)
Median V20 bone marrow (%)	69 (±8)
Median V20 bone marrow (cc)	884 (±121)
Median V10 bone marrow (%)	81 (±8.6)
Median V10 bone marrow (cc)	1042 (±206)
Median V5 bone marrow (%)	85 (±8.7)
Median V5 bone marrow (cc)	1082 (±218)
Proportion of patients receiving V10>90% bone marrow	14%
Proportion of patients receiving V 40>35% bone marrow	11.6%

EBRT, External beam radiotherapy; BT, brachytherapy; Dmax, Maximum dose; cc, cubic centimetre; V45, volume receiving 45 Gray; V40, volume receiving 40 Gray; V30, volume receiving 30 Gray; V20, volume receiving 20 Gray; V15, volume receiving 15 Gray; V10, volume receiving 10 Gray; V5, volume receiving 5 Gray

**Table 3.** Showing comparison in toxicity profile between NACT and non NACT group. (Statistically significant values ≤0.05 are shown in bold.)

	NACT cohort	Non NACT cohort	*p*-value
Leukopenia ≥ Grade II	7 (58%)	17 (55%)	0.83
Leukopenia ≥ Grade III	7 (58%)	6 (19%)	**0.01**
Neutropenia ≥ Grade II	9 (75%)	8 (25%)	**0.003**
Neutropenia ≥ Grade III	7 (58%)	3 (9.6%)	**0.002**
Thrombocytopenia ≥ Grade II	5 (42%)	4 (13%)	**0.04**
Thrombocytopenia ≥ Grade III	2 (17%)	1 (3%)	0.12
Any ≥ Grade II HT	10 (83.3%)	18 (58%)	**0.11**
Any ≥ Grade III HT	8 (67%)	7 (22.6%)	**0.007**
Acute vomiting > Grade II	5 (45.5%)	5 (18%)	**0.07**
Diarrhoea ≥ Grade II	3 (25.0%)	4 (12.9%)	0.33
Diarrhoea ≥ Grade III	2(16.7%)	3 (9.7%)	0.40
Acute Grade II or higher GI	6 (50%)	9(29%)	0.19
Acute Grade III GI	2 (16.7%)	3 (9.7%)	0.52
Any grade III or higher toxicity (HT/GI)	8 (66.7%)	9 (29%)	**0.02**


HT, haematological toxicity; GI, gastrointestinal

**Table 4.** Showing dose-volume parameters used for ROC analysis and number of patients with dosimetry exceeding the analytical parameter.

Dose-volume parameter	Number exceeding parameter
Bone marrow	
V10 > 75%	34
V10 > 90%	6
V20 > 75%	11
V20 >65%	27
Bowel	
V30 >500cc	39
V40 >200cc	39
V40 >500cc	18
V45 >200cc V45 >300cc	26 19

**Table 5.** Showing studies reporting experience with extended field intensity modulated radiotherapy (EF-IMRT).

Author and year	Study design	No. of patients	Treatment	PALN dose (Gy)	Median FU	OS	LRC	DM	Acute toxicity	Late toxicity
Chung *et al*. [14]	Prospective	63	EF-IMRT+ conc. CDDP+ BT+ Adj. CDDP+5FU	50.4	36 months	5 years 77%	5-year 86%	11%	G3 GI—2% HT—10%	G3/4—6%
Gerszten *et al*. [15]	Retrospective	22	EF-IMRT+ conc. CDDP+ BT	55	-	-	-	-	G2 GI, GU, HT- 9.5, 9.5 and 14.3%, respectively. G3—0	-
Beriwal *et al*. [3]	Retrospective	36	EF-IMRT+ conc. CDDP+ BT	55–60	18 months	2 years 65%	2-yr 80%	25%	Grade >3 GI, GU, HT 2, 2 and 27%, respectively	≥ G3—10%
Du *et al*. [16]	Prospective (EF-IMRT vs conc EFRT)	60	3# NACT + EFRT + BT	58–68 (EF-IMRT), 45-50Gy conv EFRT	28 months	3-year 36.4% EF-IMRT versus 15.6% conv EFRT (*p* = 0.016)	-	-	G3 HT, GI (IMRT versus conv) 3.6% vesrus 18.8%, *p* = 0.005; 3.6% versus 18.8%, *p* = 0.005, respectively	IMRT versus conv 0 versus 18.8% (*p* = 0.0001)
Jensen *et al*. [17]	Retrospective	21	EF-IMRT+ conc CDDP+ BT	45–50	22 months	1.5 years OS 59.7%	90.5%	42.9%	≥G2 HT, GU, GI 95.2%, 42.9%, and 9.5%, respectively	≥G3 GU—4.8% GI—0
Vargo *et al*. [18]	Retrospective	61	EF-IMRT+ conc CDDP+ BT	55	29 months	3 years 69.1	3 years 59.8	23%		≥G3—4%
Zhang *et al*. [19]	Retrospective	45	EF-IMRT+ conc. CDDP + BT	50.4	28 months	84.4% at last FU	95.2% at last FU	30.9%	≥G3 GI—7% GU—2% HT—21%	G3—6.7%
Yoon *et al*. [20]	Retrospective	90	EF-IMRT+ conc. CDDP based + BT	50.4	55 months	5 years 62.6%	-	36.7%	≥G3—3%	-
Liu *et al*. [21]	Prospective	48	EF-IMRT+ conc. Nedaplatin + BT	55–60	12 months	2 years 69.7%	-	25%	≥HT—50% GI—4.2%	4.2%
Present study	Retrospective	43	(±NACT) EF-IMRT+ conc. CDDP based + BT	45–55	12 months	2 years 89%	2 years 84%	19%	≥G3 HT, GI 22.6%, 9.7%, respectively	-

CDDP, cis-diamminedichloridoplatinum; BT, brachytherapy; OS, overall survival; LRC, locoregional control; conc, concurrent; mo, months; conv, conventional; 5FU, 5 flurouracil
